# Post-transcriptional regulation of Rbm5 expression in undifferentiated H9c2 myoblasts

**DOI:** 10.1007/s11626-015-9976-x

**Published:** 2015-12-10

**Authors:** Julie J. Loiselle, Sarah J. Tessier, Leslie C. Sutherland

**Affiliations:** Biomolecular Sciences Program, Laurentian University, 935 Ramsey Lake Road, Sudbury, ON P3E 2C6 Canada; AMRIC, Health Sciences North, 41 Ramsey Lake Road, Sudbury, ON P3E 5J1 Canada; Department of Biology, Laurentian University, 935 Ramsey Lake Road, Sudbury, ON P3E 2C6 Canada; Department of Chemistry and Biochemistry, Laurentian University, 935 Ramsey Lake Road, Sudbury, ON P3E 2C6 Canada; Division of Medical Sciences, Northern Ontario School of Medicine, Laurentian University, 935 Ramsey Lake Road, Sudbury, ON P3E 2C6 Canada; Department of Medicine, Division of Medical Oncology, University of Ottawa, Ottawa, ON Canada

**Keywords:** Rbm5, Rbm10, H9c2, Regulation, Differentiation

## Abstract

We previously examined the expression of Rbm5 during myoblast differentiation and found significantly more protein in the early stages of skeletal myoblast differentiation than during the later stages. We decided to determine if this elevated level was necessary for differentiation. Our hypothesis was that if high levels of Rbm5 protein expression were necessary for the initiation of skeletal myoblast differentiation, then inhibition of expression would prevent differentiation. Our long-term objective is to inhibit Rbm5 expression and examine the effect on H9c2 differentiation. Towards this end, stable knockdown clones and transient knockdown populations were generated. Expression analyses in H9c2 myoblasts demonstrated significant Rbm5 messenger RNA (mRNA) inhibition but, surprisingly, no effect on RBM5 protein levels. Expression of the Rbm5 paralogue Rbm10 was examined in order to (a) ensure no off-target knockdown effect, and (b) investigate any possible compensatory effects. RBM10 protein levels were found to be elevated, in both the clonal and transiently transfected populations. These results suggest that myoblast RBM5 expression is regulated by a process that includes RNA sequestration and/or controlled translation, and that (a) RBM5 function is compensated for by RBM10, and/or (b) RBM5 regulates RBM10 expression. We have developed a model to describe our findings, and suggest further experiments for testing its validity. Since upregulation of Rbm10 might compensate for downregulated Rbm5, and consequently might mask any potential knockdown effect, it could lead to incorrect conclusions regarding the importance of Rbm5 for differentiation. It is therefore imperative to determine how both RBM5 and RBM10 protein expression is regulated.

## Introduction

In general, the expression of RNA-binding motif domain protein 5 (RBM5) is highest in cells that have reduced proliferation such as aging cells (Geigl *et al.*^2004^), dormant seeds (Sugliani *et al.*^2010^), and in adult thymus compared to fetal liver (Drabkin *et al.*^1999^), and lowest in highly proliferating cells, e.g., most cancers such as non-small cell lung cancers (Oh *et al.*^2002^), vestibular schwannomas (Welling *et al.*^2002^), prostate cancers (Zhao *et al.*^2012^), stage III serious ovarian carcinomas (Kim *et al.*^2010^), pancreatic cancers (Peng *et al.*^2013^), and biliary tract cancers (Miller *et al.*^2009^). In fact, RBM5 was shown to be one of nine genes down regulated in metastasis as part of the 17-gene signature associated with metastasis in various solid tumor types (Ramaswamy *et al.*^2003^; Qiu *et al.*^2004^). The triggers for these expression fluctuations are unknown; however, using cancer cell lines, some of the mechanisms by which RBM5 expression can be regulated have been identified. For instance, RBM5 can be downregulated at the transcriptional level by a process that involves the read-through of polymerase from the upstream RBM6 gene and the consequent generation of “transcription-induced chimeras” (Wang *et al.*^2007^). Changes in RBM5 expression also occur via the regulation of alternative splicing, a role played by human epidermal growth factor receptor 2 (Rintala-Maki *et al.*^2007^) and potentially, the antisense non-coding RBM5-related factor, LUST (Rintala-Maki and Sutherland 2009). Post-transcriptionally, RBM5 can be differentially phosphorylated (Shu *et al.*^2007^).

Changes in RBM5 expression levels are associated with changes in both the expression level and the alternative splicing of downstream transcripts. For example, overexpression of RBM5 in the human leukemic cell line CEM-C7 was associated with altered expression of 35 genes, including cyclin-dependant kinase 2 (CDK2) and signal transducer and activator of transcription 5B (Stat5b), which are involved in processes such as G1/S transition and apoptosis, respectively (Mourtada-Maarabouni *et al.*^2006^). Knockdown of RBM5 was associated with altered expression of many genes in a number of different cell lines (a normal lung epithelial cell line (BEAS-2B), a normal breast epithelial cell line (MCF-10A) and three different lung cancer cell lines with varying RBM5 expression levels (A549, Calu-6 and NCI-H1299), notably increasing the expression of genes involved in cell adhesion, migration, and motility, all processes important to metastasis (Oh *et al.*^2010^). In addition, in the MCF-7 breast adenocarcinoma cell line, RBM5 and tumor necrosis factor alpha (TNF-α) expression were shown to be positively correlated, TNF-α being an important apoptosis regulatory factor (Wang *et al.*^2012^). RBM5 also regulates alternative splicing of pre-messenger RNAs (mRNAs) involved in apoptosis (exclusion of caspase-2 exon 9 (Fushimi *et al.*^2008^) and FAS receptor exon 6 (Bonnal *et al.*^2008^)), seed maturation (the inclusion of an ABIα/β exon (Sugliani *et al.*^2010^), muscular dystrophy (exclusion of dystrophin exons 40 and 72 (O’Leary *et al.*^2009^) and immunoglobulin diversification (exclusion of activation-induced cytidine deaminase exon 4 (Jin *et al.*^2012^).

Is it important to note that RBM5 shares highest homology with another RBM protein, RNA-binding motif domain protein 10 (RBM10) (Sutherland *et al.*^2005^). In fact, RBM5 and RBM10 are approximately 50% homologous at the transcript level in both human and rat. Also, endogenous RBM5 and RBM10v1 protein expression levels have been shown to be significantly positively correlated in primary breast cancer samples (Rintala-Maki *et al.*^2007^).

Similar to RBM5, RBM10 has been shown to influence the alternative splicing of many genes (Behzadnia *et al.*^2007^; Bechara *et al.*^2013^; Wang *et al.*^2013^; Zheng *et al.*^2013^; Inoue *et al.*^2014^). RBM10 is also important for the regulation of apoptosis and proliferation (James *et al.*^2007^; Wang *et al.*^2012^). Very little is known in regards to the importance of RBM5 and RBM10 in muscle differentiation; however, (a) both Rbm5 and Rbm10 are downregulated in H9c2 skeletal muscle differentiation (Loiselle and Sutherland 2014) and highly expressed in skeletal and cardiac muscle (Drabkin *et al.*^1999^; Johnston *et al.*^2010^), (b) RBM5 influences the alternative splicing of dystrophin, an important muscle protein (O’Leary *et al.*^2009^), and (c) RBM10 is important to spermatid differentiation (O’Bryan *et al.*^2013^).

In our previous study, we identified the rat H9c2 myoblast differentiation model as a suitable function-based muscle model in which to study Rbm5 and Rbm10 (Loiselle and Sutherland 2014). Our long-term objective is to determine the importance of Rbm5 to myoblast differentiation. In the study described herein, we manipulated Rbm5 expression levels in undifferentiated myoblasts, in order to characterize expression prior to differentiation. The effects of knockdown and overexpression of Rbm5 on Rbm10 mRNA and protein expression levels were also examined, to rule out off-target knockdown effects and to determine if changes in Rbm5 expression effected the expression of Rbm10, prior to differentiation. The interesting observations that were made have been incorporated into a model that will be tested in future experiments.

## Materials and Methods

### Cell culture.

Cells were grown as previously described (Loiselle and Sutherland 2014).

### Stable knockdown.

At 24 h prior to transfection, cells were passed in 100-mm plates (Sarstedt, Montreal, Canada) and as such that they would be approximately 35% confluent at the time of transfection. Twenty-four hours following appropriate plating, 12 μl of Lipofectamine 2000 transfection reagent (Life Technologies, Burlington, Canada) was mixed with 1.5 ml of Opti-MEM reduced serum media (Life Technologies) with GlutaMAX (Life Technologies), and incubated at room temperature for 5 min. The appropriate small hairpin RNA (shRNA) construct (12 μg) was also mixed with 1.5 ml of Opti-MEM reduced serum media with GlutaMAX, and incubated at room temperature for five minutes. Control samples were transfected with CSHCTR001-nU6 shRNA scrambled control (GeneCopoeia, Rockville, MD). Rbm5 knockdown samples were transfected with both MSH039757-1 and MSH039757-6 Rbm5-specific shRNAs (6 μg of each) (GeneCopoeia) (Table 1). The Rbm5-specific shRNAs were 100% homologous to both rat and mouse Rbm5 sequence. Following, the Lipofectamine 2000 + Opti-MEM and shRNA + Opti-MEM solutions were mixed together and incubated at room temperature for 35 min. The transfection solution was then added to the normal, serum-containing media on the cells. Selection began 24 h post-transfection by treating cells with 1 μg/ml of puromycin. Cells were then cultured in antibiotic-containing media to select for successful transfectants for at least 28 days following transfection and until they filled a 100-mm plate (Sarstedt).

**Table 1.** Small interfering RNA Rbm5 knockdown oligonucleotides

Type	Name	Sequence	Location	Homology to Rbm10
shRNA	CSHCTR001-nU6		Scrambled	
	MSH039757-1	5′ GTAGTGGAAGATATGGTTC 3′	Exon 3	(12/19) 63%
	MSH039757-6	5′ GAGCGATATTCGAGAAATG 3′	Exon 4/5	(12/19) 63%
siRNA	Trilencer-27mer universal scrambled negative control		Scrambled	
	ON-TARGET RBM5 duplex siRNA	5′ GAGCGATATTCGAGAAATG 3′	Exon 4/5	(12/19) 63%

### Transient knockdown.

Twenty-four hours prior to transfection, cells were passed to 6-well plates (Sarstedt) so as to be 40% confluent at the time of transfection. Twenty-four hours after appropriately plating cells, 5 μl of Lipofectamine 2000 transfection reagent (Life Technologies) was added to 245 μl of Opti-MEM reduced serum media (Life Technologies) with GlutaMAX (Life Technologies), and incubated at room temperature for 5 min. The appropriate small interfering RNA (siRNA) or shRNA was also added to 245 μl of Opti-MEM reduced serum media with GlutaMAX, and incubated at room temperature for 5 min. The first transient knockdown experiment was performed with siRNA. The siRNA used for the control sample was Trilencer-27mer universal scrambled negative control siRNA duplex (OriGene, Rockville, MD). For Rbm5 knockdown samples, custom on-target RBM5 duplex siRNA (Dharmacon, Thermo Fisher Scientific, Ottawa, Canada) was used (Table 1). The second and third transient knockdown experiments were performed with shRNAs (same constructs used for stable knockdown experiments). Thus, control samples were transfected with the scramble control CSHCTR001-nU6, and Rbm5 transient knockdown experiments two and three were transfected with MSH039757-1 and MSH039757-6 Rbm5-specific shRNAs, respectively (GeneCopoeia) (Table 1). siRNAs and shRNAs had a final concentration of 10 nM when administered to cells. Following a 5-min incubation, the Lipofectamine 2000 + Opti-MEM and siRNA + Opti-MEM solutions were mixed together and incubated at room temperature for 20 min. Next, the transfection solution was added to the normal, serum-containing media on the cells. Medium was not changed after addition of transfection solution, and cell pellets were collected at 72 h post-transfection.

### Transient overexpression.

The plasmid used for the transient overexpression of RBM5 was pcDNA3. RBM5 (Rintala-Maki and Sutherland 2004), with pcDNA3 empty vector as the negative control. Note that RBM5 sequence in the vector was human, which has approximately 80% homology with rat. Cells were passaged such that a confluency of 35% would be obtained on the day of the experiment. At that point, 12 μg of the respective pcDNA3 plasmid and 18 μl of Lipofectamine 2000 transfection reagent (Life Technologies) were each mixed separately with 1.5 ml of Opti-MEM reduced serum medium (Life Technologies) with GlutaMAX (Life Technologies). After a 5-min incubation at room temperature, the DNA + Opti-MEM and Lipofectamine 2000 + Opti-MEM mixtures were mixed together, and incubated at room temperature for 20 min. Following this, the transfection mixture was added to the cells. The medium was not changed after addition of the transfection mixture, and cell pellets were collected at 72 h post-transfection.

### RNA expression analysis.

RNA extraction, reverse transcription, and end-point semi-quantitative PCR were performed as previously described (Loiselle and Sutherland 2014), except in this case, 24 amplification cycles were used for glyceraldehyde 3-phosphate dehydrogenase (Gapdh), and 38 cycles for Rbm5 and Rbm10 (Table 2).

**Table 2.** Primers for end-point PCR

Gene name	Accession no.	Primer sequence (5′ to 3′)	Amplicon length (bp)	Annealing temp. (°C)
*Actb*	NM_031144	F: TGAGCGCAAGTACTCTGTGTGGAT	129	62
		R: TAGAAGCATTTGCGGTGCACGATG		
*Gapdh*	BC059110	F: ACCACAGTCCATGCCATCAC	452	58
		R: TCCACCACCCTGTTGCTGTA		
*Rbm5*	BC166477	F: ATGGGTTCAGACAAAAGAG	520	55
		R: GCATTGCAATGTGCTTTCCTTGA		
*Rbm10*	F1LWMO	F: ATTGGCTCCCGTCGAACTAACAGT	916 (10v1)	63
		R: ACTTCTCTCGGCGCTTGAAGTTCT	682 (10v2)	

*F* forward primer (5′ primer), *R* reverse primer (3′ primer)

Densitometric analysis was performed using AlphaEase FC software (Alpha Innotec, Kasendorf, Germany). Rbm5 and Rbm10 mRNA expression values were first normalized to the 300-bp ladder band to account for gel exposure differences between replicates, then to Gapdh, the reference gene used. Next, the average of the normalized expression value obtained for all technical replicates of a biological replicate was determined. This average normalized expression value was then expressed as a fold-change from the control sample of that biological replicate, and averaged for the various biological replicates. This gave the final expression value that was graphed.

### Protein expression analysis.

Protein samples were prepared as previously described (Sutherland *et al.*^2000^). Primary antibodies used were mouse anti-α-tubulin (1:10,000, sc-8035, Santa Cruz Biotechnologies Inc., Santa Cruz, CA); rabbit anti-RBM10 (1:1000, A301-006A, Bethyl Laboratories Inc/Cedarlane, Burlington, Canada); rabbit anti-RBM5 (1:2500, ab85504, Abcam, Toronto, Canada); rabbit anti-RBM5 LUCA-15UK (1:2000, non-commercially available (Sutherland *et al.*^2000^)); and rabbit anti-RBM5 SP1 and SP2 (1:1000, non-commercially available (Bonnal *et al.*^2008^)). A goat anti-mouse horseradish peroxidase (HRP)-conjugated secondary antibody (1:20,000, sc-2005, Santa Cruz Biotechnologies Inc.) and a goat anti-rabbit HRP-conjugated secondary antibody (1:10,000, sc-2004, Santa Cruz Biotechnologies Inc.) were employed. The presence of antibodies on the membrane was detected using Amersham ECL Western Blotting Detection Reagents (GE Healthcare, Mississauga, Canada) and Amersham Hyperfilm ECL (GE Healthcare).

The membranes were stripped between probing with different primary antibodies using the following mild stripping procedure: two 10-min washes in mild stripping buffer (1.5% glycine, 0.1% SDS, 0.1% Tween 20, pH 2.2); two 10-min washes in phosphate-buffered saline (1× PBS, pH 7.4, Life Technologies); and finally, two 5-min washes with Tris-buffered saline with Tween 20 (TBS-T). Densitometric analysis was performed on the resulting blots using AlphaEase FC software. The resulting RBM5 and RBM10 expression values were first normalized to α-tubulin, the reference gene used. Normalized expression values were then expressed as fold-change from the control sample for that biological replicate (control sample present on each gel), and the average of the technical replicates for each biological replicate determined. Following, the average across biological replicates was determined and graphed.

## Results and Discussion

### Rbm5 mRNA knockdown has no effect on Rbm5 protein levels.

**Figure 1.**
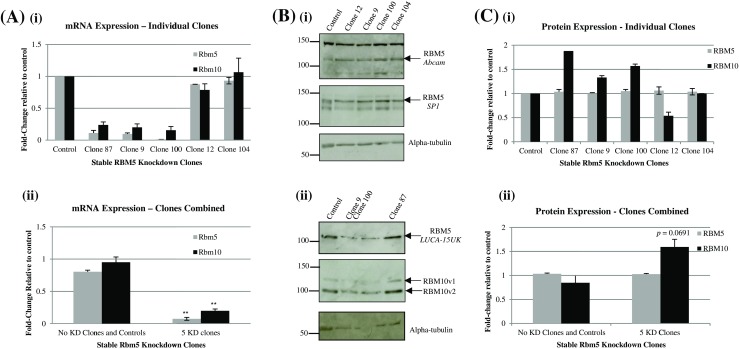
Rbm5 and Rbm10 expression in Rbm5 shRNA stably transfected H9c2 clones. (*A*) RT-PCR results for Rbm5 and Rbm10 expression in the various RBM5 knockdown (KD) clones, individually from technical duplicates (*i*) and pooled (*ii*). Gapdh was used as reference gene. (*B*) Representative raw Western blot protein expression data for RBM5 and RBM10 using one anti-RBM10 antibody and various anti-RBM5 antibodies (antibody name or manufacturer indicated on the right of the blots). Precision Plus ladder (BioRad) was used, and ladder values refer to weight in kilodalton (kDa). (*C*) Densitometric analysis of protein expression in the various clones, individually (from technical duplicates for each clone except clone 87 and 104 in regards to RBM10 expression) (*i*) and pooled (*ii*). Results were normalized to alpha-tubulin. In the pooled figures (*Aii*, *Cii*), “No KD Clones and Controls” represents results from the scrambled control as well as clones which did not show at least 70% knockdown of Rbm5 at the RNA level (clones 12 and 104), whereas “5 KD Clones” represent results from those clones that did show mRNA knockdown of Rbm5 (clones 87, 9, and 100). Values represent mean ± standard error (SE). *Asterisk* placed directly above a *bar* indicates value is statistically different from control as determined by unpaired *t* test (**indicates *p* < 0.01).

**Figure 2.**
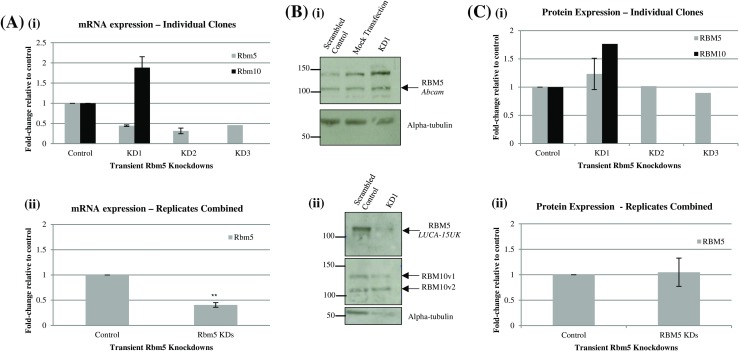
Rbm5 and Rbm10 expression in Rbm5 shRNA/siRNA transiently transfected H9c2 cells. (*A*) RT-PCR results for Rbm5 and Rbm10 expression in the various transient RBM5 knockdown (*KD*) experiments, individually (from technical duplicates, except KD3 (one technical replicate)) (*i*) and pooled (*ii*). Gapdh was used as reference gene. (*B*) Representative raw Western Blot protein expression data for RBM5 and RBM10 using one anti-RBM10 antibody and various anti-RBM5 antibodies (antibody name or manufacturer indicated on the *right* of the blots). Precision Plus ladder (BioRad) was used, and ladder values refer to weight in kDa. (*C*) Densitometric analysis of protein expression in the various KD experiments, individually from one technical replicate (two for KD1 expression of RBM5) (*i*) and pooled (*ii*). Results were normalized to alpha-tubulin. Values represent mean ± SE. *Asterisk* placed directly above a *bar* indicates value is statistically different from control as determined by unpaired *t* test (** indicates *p* < 0.01).

One hundred seventeen Rbm5 shRNA transfected H9c2 clones were obtained following 28 or 29 days of selection in puromycin. All 117 clones were screened for Rbm5 mRNA expression. Three clones (clones 87, 9, and 100) with significant inhibition of Rbm5 mRNA expression (>80%), compared to the scrambled control, were chosen for further analysis, along with two clones (clones 12 and 104) with no visible knockdown (Fig. 1*A*). Surprisingly, Rbm5 protein levels were not significantly inhibited in any of the Rbm5 knockdown clones (Fig. 1*B, C*). To rule out any possible clonal effect that could account for abnormal regulation of protein expression, three transient transfections were performed. The first transient transfection used siRNA-specific to Rbm5 sequence (but different from one of the shRNA sequences used) (KD1). The second and third transient transfections used the shRNA from the stable knockdown experiments, MSH039757-1 and MSH039757-6, respectively. mRNA knockdowns ranged between 55% and 70% (Fig. 2*A*), but, once again, there was no decrease in Rbm5 protein expression levels (Fig. 2*B, C*). To ensure that antibody affinity was not an issue, three different anti-RBM5 antibodies were used in the stable knockdown analysis, and two in the transient knockdown work (Figs. 1*B* and 2*B*).

### Rbm5 knockdown correlates with increased Rbm10 protein levels.

Since rat Rbm10 has 57% homology with rat Rbm5, to ensure no off-target effect of the theoretically Rbm5-specific sh/siRNAs on Rbm10 expression, Rbm10 expression was also examined in the knockdowns. Both shRNA and siRNA sequences were chosen to limit the potential for off-target effects on Rbm10: Rbm5 shRNA and siRNA sequences were 19-mers with seven mismatches to rat Rbm10, meaning they had 63% homology. In the clones, at the RNA level (Fig. 1*A*), Rbm10 expression significantly decreased in of all the clones with the most significant Rbm5 RNA knockdown (clones 87, 9, and 100). At the protein level (Fig. 1*C*), Rbm10 expression was surprising increased, but only in the Rbm5 clones with the most significant Rbm5 RNA knockdown. Additionally, Rbm10 protein expression was unexpectedly decreased in clone 12, which had shown no change in Rbm5 mRNA expression levels as a result of knockdown. In the transient Rbm5 knockdown, Rbm10 mRNA and protein expression were increased almost twofold, compared to the scrambled control (Fig. 2*A, C*).

### Rbm5 overexpression does not correlate with decreased Rbm10 protein levels.

**Figure 3.**
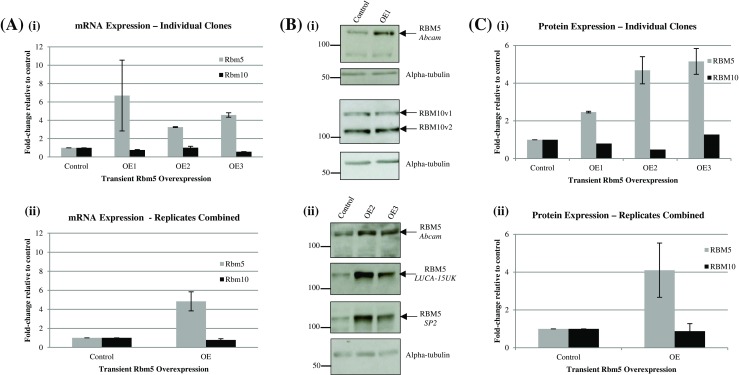
Rbm5 and Rbm10 expression in Rbm5 transiently overexpressed H9c2 cells. (*A*) RT-PCR results for Rbm5 and Rbm10 expression in the various transient RBM5 overexpression (*OE*) experiments, individually from technical duplicates (*i*) and pooled (*ii*). Gapdh was used as reference gene. (*B*) Representative raw Western Blot protein expression data for RBM5 and RBM10 using one anti-RBM10 antibody and various anti-RBM5 antibodies (antibody name or manufacturer indicated on the right of the blots). Precision Plus ladder (BioRad) was used, and ladder values refer to weight in kDa. (*C*) Densitometric analysis of protein expression in the various OE experiments, individually from technical duplicates for RBM5 expression and one technical replicate for RBM10 expression (*i*) and pooled (*ii*). Results were normalized to alpha-tubulin. Values represent mean ± SE. Statistical significance was evaluated using an unpaired *t* test for each sample compared to their respective control.

Since inhibition of Rbm5 correlated with increased expression of RBM10 in both the stable and transient knockdowns, we sought to determine if the reverse were true, and overexpression of Rbm5 correlated with decreased Rbm10 expression. Transient overexpression of RBM5 protein from the human complementary DNA (cDNA) sequence (which has approximately 80% homology with rat) was confirmed with three different anti-RBM5 antibodies (Fig. 3*B*), but Rbm10 protein expression levels remained unchanged, compared to the scrambled control transfectants (Fig. 3*B,C*).

Based on our findings, a number of conclusions can be drawn. Firstly, only a small quantity of Rbm5 mRNA is translated. Secondly, regulation of Rbm5 protein expression in H9c2 myoblasts has unique characteristics. Thirdly, decreased Rbm5 mRNA levels regulate Rbm10 protein expression. In the following sections, we discuss each of these conclusions, and present a model that depicts them.

### Only a small quantity of Rbm5 mRNA is translated.

Knockdown of Rbm5 mRNA in either stable or transient transfections is not reflected at the protein level. Lack of a positive correlation between Rbm5 mRNA and protein expression in the transient transfections could possibly relate to the fact that (a) Rbm5 protein is very stable or (b) the mRNA was not inhibited sufficiently to have an effect. When these transient data are combined with the lack of correlation between Rbm5 mRNA and protein expression in the stable clones, the data suggest that unchanged Rbm5 protein expression levels was not due to (a) any possible clonal effect in the stable clones or (b) Rbm5 protein stability. A precedented explanation for the lack of correlation between Rbm5 mRNA and protein expression levels in the knockdown experiments is that only a fraction of endogenous Rbm5 mRNA is actually translated into protein. The shRNA was able to degrade up to 90% of the Rbm5 mRNA in the stable clones, but the ∼10% that was left might be all that is normally translated in the wild-type myoblasts.

One possible reason to account for the fact that only a small portion of Rbm5 mRNA may be translated is that messenger ribonucleoproteins (mRNPs) may be involved in sequestering the majority of Rbm5 mRNA in H9c2 cells. Precedent for this occurs in *Xenopus* oogenesis, where 80% of maternal mRNAs are sequestered in mRNP storage particles, and translation is inhibited until specific time-points during early embryogenesis when the mRNAs are recruited to ribosomes and finally translated (Spirin 1966; Tafuri and Wolffe 1993). A second precedent occurs in P19 murine embryonic carcinoma cell differentiation, where the composition of mRNP-sequestered mRNAs changes following exposure to differentiation-inducing stimuli (Tenenbaum *et al.*^2000^). Furthermore, in satellite cells, transcripts of Myf5, an important regulator of myogenesis, have been shown to be sequestered in mRNP granules. Upon activation of the satellite cell, these granules dissociate, leading to liberation of myogenic factor 5(Myf5) transcripts and consequently higher levels of Myf5 (Crist *et al.*^2012^). This regulatory mechanism thus allows quiescent satellite cells to transcribe Myf5 without activating differentiation. A similar mechanism could be occurring in the H9c2 cells, which would explain not only why a 90% knockdown of Rbm5 mRNA is not reflected at the protein level, and why the changes in Rbm5 protein levels during cardiac differentiation were not positively correlated with changes in Rbm5 mRNA levels (i.e., during differentiation, it is not the total amount of Rbm5 mRNA in the cell that is important but the amount that is not sequestered, and thus available for translation) (Loiselle and Sutherland 2014).

In the overexpression experiments, exogenous Rbm5 mRNA was translated. If our sequestering hypothesis is correct, this result suggests that either (a) the cell could distinguish between exogenously and endogenously transcribed Rbm5 transcript, or (b) there was a finite quantity of Rbm5 message that could be sequestered, a quantity that might be regulated by levels of endogenous Rbm5 protein or Rbm10 mRNA/protein levels.

### Regulation of Rbm5 protein expression in H9c2/myoblasts has unique characteristics.

Correlations between RBM5 expression at both the mRNA and protein levels have been examined in breast (Oh *et al.*^2002^; Rintala-Maki *et al.*^2007^), lung (Liang *et al.*^2012^), and pancreatic (Peng *et al.*^2013^) non-tumor and tumor tissue, and various cell lines including A549 (lung adenocarcinoma) (Oh *et al.*^2010^; Li *et al.*^2012^), Calu-6 (possibly lung carcinoma) (Oh *et al.*^2010^), NCI-H1299 (non-small cell lung carcinoma) (Oh *et al.*^2010^), U2OS (osteosarcoma) (Kobayashi *et al.*^2011^), PC-3 (prostate adenocarcinoma) (Zhao *et al.*^2012^), BEAS-2B (immortalized human bronchial epithelial cells) (Oh *et al.*^2010^), HEK293 (human embryonic kidney cells) (Fushimi *et al.*^2008^), MCF-10A (immortalized epithelial cells derived from human fibrocystic mammary tissue) (Oh *et al.*^2010^), and those of various mantle cell and follicular lymphomas (Weinkauf *et al.*^2007^): a positive correlation between mRNA and protein expression levels was consistently observed. Only in non-tumor breast tissue was a positive correlation between RBM5 mRNA and protein expression not observed (Rintala-Maki *et al.*^2007^). Therefore, the mechanism suggested earlier in which only a percentage of Rbm5 mRNA is translated, and the rest is sequestered (perhaps in mRNPs) may be a restricted phenomenon that occurs in, for example, particular cell types or in cells with certain growth characteristics, including rat myoblasts.

### Decreased Rbm5 mRNA levels regulate Rbm10 protein expression.

It was interesting to note that, despite unchanged levels of Rbm5 protein, Rbm10 protein levels went up. This observation was particularly interesting in view of the fact that Rbm10 mRNA levels significantly decreased in the stable knockdowns. Any potential off-target effect of Rbm5 shRNA on Rbm10 was considered highly unlikely once the elevated levels of Rbm10 protein were observed. The results suggest a complex regulatory mechanism linking degradation of Rbm5 mRNA with Rbm10 protein expression. It is important to note that another group has also recently reported that decreased expression of Rbm5 correlated with increased levels of Rbm10. Their study was performed in injured mouse brain homogenates (Jackson *et al.*^2015^).

### Model.

Based on the results of the Rbm5 knockdown and overexpression experiments, we hypothesize that the majority of Rbm5 transcripts are sequestered, possibly in mRNPs, and unavailable for translation. Release of sequestered Rbm5 transcripts would occur at certain points during differentiation, as required. Therefore, this could be the post-transcriptional mechanism regulating Rbm5 expression throughout H9c2 skeletal and cardiac differentiation suggested by our group previously (Loiselle and Sutherland 2014).

**Figure 4.**
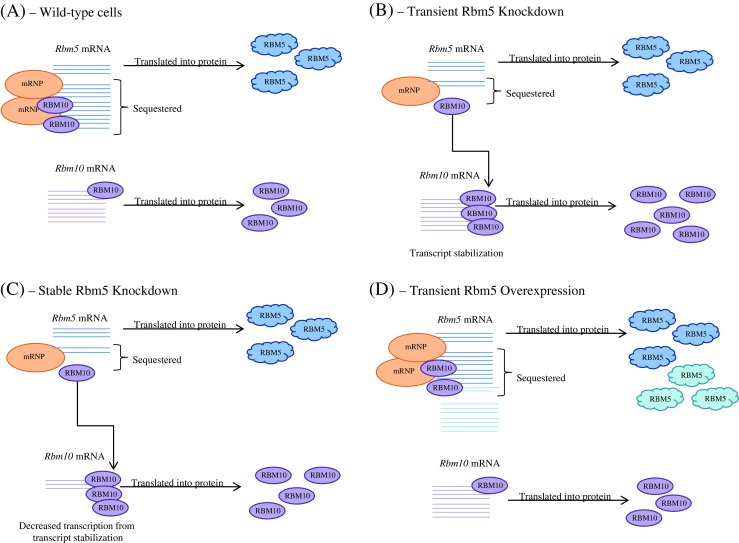
Model representing the effects of varying levels of Rbm5 mRNA on the expression of RBM5 and RBM10. Rbm5 and Rbm10 expression in H9c2 wild-type cells (*A*), upon transient Rbm5 knockdown (*B*), upon stable Rbm5 knockdown (*C*), and upon transient Rbm5 overexpression (*D*). RBM5 protein is represented by *blue clouds*, with *lighter blue* representing overexpressed protein, and RBM10 protein is represented by *purple ovals*. *Orange mRNP ovals* represent complex mRNP particles with various components. For in-depth model description, refer to text.

**Figure 5.**
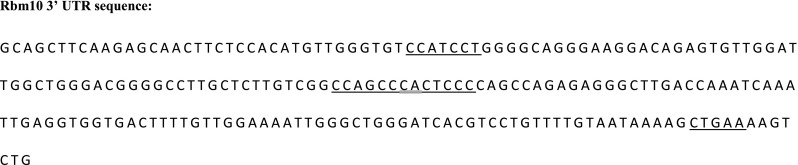
RBM10 binding sites in the Rbm10 3′ UTR. Segments of the top consensus motifs for RBM10 binding sites, as determined by Bechara *et al.* (2013), *underlined* at their respective locations in the Rbm10 3′ UTR. *Double underlined* sequences are part of the motifs on each side. All sequences have two mismatches compared to the *top* previously identified motif, except the most 3′- motif which is a complete match.

We postulate that two things are occurring in H9c2 cells regarding RBM10. First, that RBM10 is a component of the mRNP complexes sequestering Rbm5 mRNA in H9c2 cells (Fig. 4*A*), as RBM proteins have been shown to play important roles in these structures, as previously reviewed (Hieronymus and Silver 2004). Secondly, that RBM10 can bind the 3′ UTR of its own mRNA transcript and thereby increase its own stability. The rat homologue of RBM10, S1-1, has already been shown to bind the 3′ UTR of angiotensin II receptor type 1 (AT1), stabilizing the message, and ultimately decreasing transcription (Mueller *et al.*^2009^). Furthermore, previously identified RBM10 binding sequences are located within the Rbm10 3′ UTR (Fig. 5) (Bechara *et al.*^2013^). It is important to note that Bechara et al. demonstrated that even 2/7 mismatches from the *top* selected motifs still enabled good RBM10 binding.

In our model, when Rbm5 mRNA is decreased and its associated mRNP complexes disassembled, RBM10 protein would be released into the cell. This RBM10 would be free to bind the 3′ UTR of Rbm10 mRNA, stabilizing the transcript (Fig. 4*B*). Initially, this may lead to increased levels of Rbm10 mRNA and protein, which is what we observed in our transient Rbm5 knockdown experiments (Fig. 2). Ultimately, however, this stabilization may lead to decreased transcription of Rbm10, as has been shown upon rat RBM10 stabilization of AT-1 transcript (Mueller *et al.*^2009^) (Fig. 4*C*). As a result, lower levels of Rbm10 mRNA would be expected. This is, in fact what we observed in our Rbm5 stable knockdown clones (Fig. 1).

On the other hand, upon overexpression of Rbm5, we would expect these non-physiological levels to be too high to all be sequestered in mRNP complexes, resulting in their transcription, translation, and, as a result, higher levels RBM5 (Fig. 4*D*). Furthermore, since the mRNP complexes sequestering Rbm5 would not be disrupted by this overexpression, we would expect Rbm10 mRNA and protein levels to remain constant. As shown in Fig. 3, this is what we observed experimentally; higher levels of RBM5 upon Rbm5 overexpression, but no change in Rbm10 mRNA or protein levels.

To test the validity of this model, immunoprecipitation of Rck/p54 (p-body protein and mRNP complex component) could be performed, followed by next generation sequencing of its associated RNA (RIP-Seq). This would determine if Rbm5 is among the mRNP-sequestered transcripts in H9c2 myoblasts. Furthermore, RNA-binding protein purification and identification (RaPID) could be used to determine if Rbm10 is a component of the mRNP/Rbm5 mRNA complexes. This would involve tagging Rbm5 mRNA, transfecting it into H9c2 cells, purifying the tagged transcripts, then detecting associated proteins via mass-spectrometry (Slobodin and Gerst 2010). Direct binding of Rbm10 to its own transcript could be analyzed via electromobility shift assays (EMSAs), using tagged Rbm10 3′ UTR probes. Knockdown and overexpression of Rbm10 would not be useful to test this model since it would involve directly manipulating levels of Rbm10, which may mask any effect that protein levels have on transcript expression.

## Conclusion

The results from this work suggest that Rbm5 is post-transcriptionally regulated in rat myoblasts. More specifically, our results suggest that only a small portion of Rbm5 mRNA may be translated in myoblasts, while the rest is sequestered in the cell. Results from Rbm10 mRNA and protein expression in Rbm5 knockdown and overexpression samples also suggest that Rbm10 expression is influenced by Rbm5 and by its own protein levels. This co-regulation has already been shown in neuronal cells (Jackson *et al.*^2015^), and suggests that, as in transformed cells (Sutherland *et al.*^2000^; Bonnal *et al.*^2008^; Fushimi *et al.*^2008^; Wang *et al.*^2012^; Inoue *et al.*^2014^), Rbm5 and Rbm10 may influence similar cellular processes in myoblasts. Finally, the intricate regulation of Rbm5 protein levels in H9c2 cells suggest a function of the utmost importance to myoblast differentiation, and perhaps, muscle development in general.
